# Actin Cytoskeleton Dysregulation Links Testicular and Sperm Dysfunction in Type 1 Diabetes †

**DOI:** 10.3390/ijms27167423

**Published:** 2026-08-19

**Authors:** Maria Rosaria Ambruosi, Alessandra Biasi, Serena Boccella, Ilef Romdhani, Sara Falvo, Francesca Guida, Sabatino Maione, Sergio Minucci, Massimo Venditti

**Affiliations:** 1 Dipartimento di Medicina Sperimentale, Università degli Studi della Campania “Luigi Vanvitelli”, 80138 Napoli, Italy; mariarosaria.ambruosi@unicampania.it (M.R.A.); alessandra.biasi@unicampania.it (A.B.); boccellaserena@gmail.com (S.B.); ilef.romdhani@unicampania.it (I.R.); francesca.guida@unicampania.it (F.G.); sabatino.maione@unicampania.it (S.M.);; 2 Dipartimento di Science e Tecnologie Ambientali, Biologiche e Farmaceutiche, Università degli Studi della Campania “Luigi Vanvitelli”, 81100 Caserta, Italy; sara.falvo@unicampania.it

**Keywords:** spermatogenesis, spermatozoa, F-actin, actin-binding proteins, planar cell polarity, DAAM1, cofilin, MARCKS, acrosome reaction, PKC

## Abstract

Type 1 diabetes (T1D) is a systemic metabolic disorder associated with male reproductive dysfunction. Given the pivotal role of actin cytoskeleton remodeling in spermatogenesis and sperm function, this study investigated the effects of T1D on actin-regulating pathways in rat testis and spermatozoa (SPZ). Adult Wistar rats were rendered diabetic by streptozotocin administration (65 mg/kg, i.p.). Testicular analysis revealed a reduced F-/G-actin ratio together with marked F-actin disorganization, consistent with altered actin cytoskeleton remodeling. To investigate the molecular mechanisms underlying these alterations, key regulators of actin dynamics were examined. Diabetic animals displayed impaired expression of EPS8, Fascin, N-WASP, and the ARP2/3 complex, suggesting altered regulation of actin assembly, bundling, and branching. Further analyses demonstrated dysregulation of signaling pathways governing cytoskeletal organization. Reduced levels of phosphorylated Disheveled-2, DAAM1, RhoA-GTP, and ROCK1 indicated impairment of the planar cell polarity pathway. In parallel, changes in LIMK1/cofilin phosphorylation supported abnormal regulation of actin filament turnover. Alterations in the RICTOR/PKC/MARCKS signaling pathway further highlighted defects in cytoskeletal control. Similar abnormalities were observed in mature SPZ, where altered F-actin distribution and DAAM1 localization suggested persistent cytoskeletal defects. Moreover, diabetic SPZ exhibited a reduced ability to undergo acrosome reaction, accompanied by altered MARCKS phosphorylation, highlighting defects in actin-dependent processes essential for sperm function and fertilizing capacity. These findings indicate that disruption of actin cytoskeleton dynamics may represent a major mechanism contributing to testicular and sperm abnormalities in T1D, providing new insights into the mechanisms underlying diabetes-associated male reproductive dysfunction.

## 1. Introduction

The production and differentiation of high-quality gametes rely on a series of tightly regulated events that, in males, occur within the seminiferous epithelium (SE) of the testis. This progression includes the mitotic proliferation of spermatogonia, the generation of haploid spermatids (SPT) through meiosis, their maturation into fully differentiated spermatozoa (SPZ) during spermiogenesis, and, ultimately, their release into the tubular lumen. Sertoli cells (SC) support these events by forming a polarized epithelial layer that provides structural, nutritional, and regulatory support to developing germ cells (GC) [1,2]. This complex differentiation program requires the coordinated activation and repression of numerous genes, many of which are testis-specific and exhibit tightly regulated spatial and temporal expression patterns [3,4,5,6,7].

During the epithelial cycle of spermatogenesis, GC differentiate into progressively advanced stages, which are transported by SC across the SE from the basal to the adluminal (apical) compartment, allowing SPZ spermiation. However, unlike other mammalian cells, GC lack the molecular machinery required for active locomotion and, thus, are immotile; therefore, GC, acting as “cargoes”, rely on actin microfilaments within SC that function as the “vehicle” for their transport across the SE, while microtubules serve as the “railroad tracks” that guide this movement [8,9].

Indeed, specialized actin-based cytoskeletal structures are essential components of the mammalian testis and play pivotal roles in maintaining SE organization and GC development [10]. Among these structures, the ectoplasmic specialization (ES) is a testis-specific actin-rich junction characterized by densely packed actin filament bundles positioned between the plasma membrane and cisternae of the endoplasmic reticulum. Depending on its localization, the ES can be found either between adjacent SC (basal ES) or at the SC–elongating SPT interface (apical ES) [11,12].

Actin exists in two interconvertible forms: globular, monomeric actin (G-actin) and filamentous actin (F-actin), also defined as microfilament [13]. The dynamic balance between actin microfilament polymerization and depolymerization facilitates continuous cytoskeletal remodeling, which regulates diverse cellular processes such as cell adhesion, polarity, migration, intracellular trafficking, and morphogenesis [14]. In addition, in SPZ, actin microfilament dynamics are tightly regulated during capacitation and the acrosome reaction (AR), highlighting the importance of actin turnover not only during spermatogenesis but also for the acquisition of fertilizing capacity [15].

Actin dynamics encompass multiple processes, including nucleation, polymerization, depolymerization, bundling, capping, branching, cross-linking, and severing, all of which require coordinated activity of a multitude of actin-binding proteins (ABPs) [13,15]. Among these are proteins such as epidermal growth factor receptor pathway substrate 8 (EPS8) [16] and Fascin1 (FSCN-1) [17], which contribute to the formation of highly ordered F-actin structures characteristic of the ES by facilitating actin bundling and stabilization. Conversely, nucleation-promoting factors, including Neural Wiskott–Aldrich syndrome protein (N-WASP) [18,19] and the Actin-Related Protein 2/3 (Arp2/3) complex [19], induce actin polymerization and branching, thereby enabling cytoskeletal plasticity and junction remodeling. Interestingly, in addition to ABPs, testicular actin cytoskeletal organization and remodeling are modulated by upstream signaling pathways, including the non-canonical Wnt/planar cell polarity (PCP) pathway [20,21,22,23,24]. Components of this signaling cascade, including Frizzled 5 (FZD5) receptors [25], Disheveled-2 (DVL2) [25], Disheveled-Associated Activator of Morphogenesis 1 (DAAM1) [26,27], Ras Homolog Family Member A (RhoA) [28,29] and Rho-associated protein kinase 1 (ROCK1) [30,31] and downstream effectors, including LIM kinase 1(LIMK1) [29,30,31,32] and cofilin (CFL1) [29,33], have been identified in the testis and are essential in the regulation of actin filament organization, as well as GC and SC polarity within the SE.

In addition to PCP signaling, actin remodeling is regulated by the mechanistic target of rapamycin complex 2 (mTORC2), which, through its core component Rapamycin-Insensitive Companion of mTOR (RICTOR), modulates cytoskeletal dynamics by regulating downstream kinases such as protein kinase C (PKC) [34,35,36,37]. One of its targets, the myristoylated alanine-rich C kinase substrate (MARCKS), is critical for linking actin filaments to the plasma membrane and for controlling cortical actin organization. Phosphorylation of MARCKS leads to its dissociation from the membrane, thereby affecting actin filament distribution and membrane-associated processes [38,39].

The importance of the role of ABPs and the signaling cascades regulating actin microfilament dynamics relies on the fact that their deletion, inactivation, mutation, and/or genetic variation in regulatory genes may lead to subfertility in humans and rodents [15].

In recent years, the study of ABPs and cytoskeletal dynamics in spermatogenesis has been developed based on the use of toxicant models, such as endocrine disruptors and oxidative stress (OS) inducers, including cadmium, PFOS, and the male contraceptive adjudin [15,19,40,41,42,43,44,45,46].

Among the systemic pathologic conditions associated with male reproductive dysfunction, type 1 diabetes (T1D) represents a major contributor to testicular impairment and subfertility. Indeed, many studies have demonstrated not only that T1D adversely affects the hypothalamic–pituitary–gonadal axis [47,48,49], but that chronic hyperglycemia is associated with increased OS, inflammation, and apoptosis in the SE, leading to impaired sperm quality [50,51].

Consistently, in our previous works, we confirmed the detrimental effects on testicular activity in a T1D rat model, in terms of impaired steroidogenesis, spermatogenetic progression, impaired blood-testis barrier (BTB) integrity, and induced inflammation [52]. In addition, we have also observed a potential impact of T1D-associated OS on microtubule dynamics and associated proteins in the testis and SPZ, offering new insights into the molecular mechanisms potentially contributing to male subfertility in the context of T1D [53]. Given the susceptibility of actin microfilaments and ABPs to diabetes-associated cellular stress, this study investigated the impact of T1D on actin cytoskeleton organization and remodeling in the testis by evaluating the F-/G-actin balance, selected ABPs, and their associated regulatory signaling pathways. Finally, the analysis was extended to mature SPZ under basal conditions and following in vitro AR induction to evaluate whether T1D-associated alterations of the actin cytoskeleton persist beyond spermatogenesis and affect sperm functional competence.

## 2. Results

### 2.1. Effects of T1D on Actin Cytoskeleton Organization

**Figure 1:**
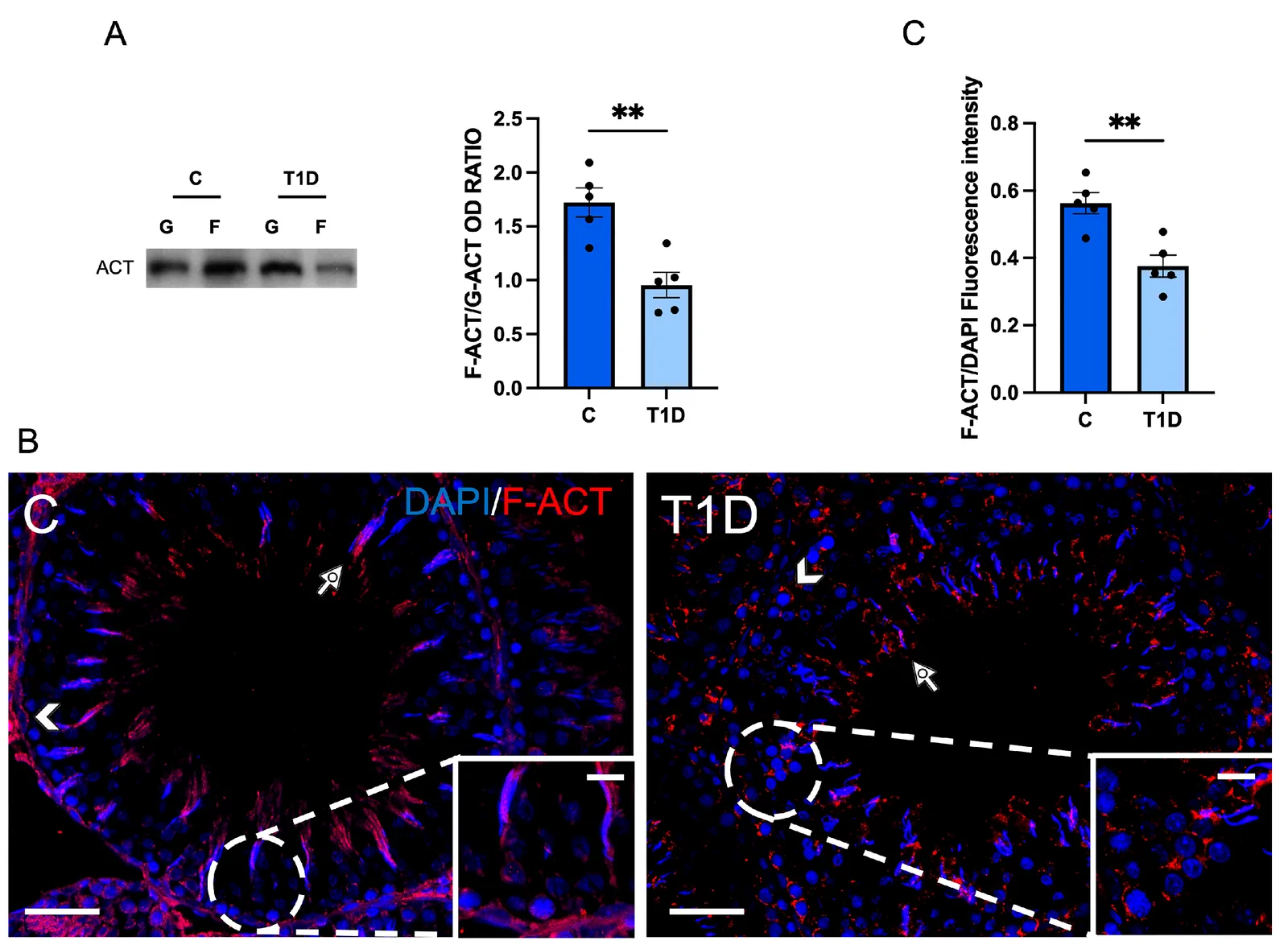
Analysis of F-actin (F-ACT) organization in control and type 1 diabetes (T1D) rat testes. (**A**) Biochemical fractionation of globular (G)- and filamentous (F)-actin followed by Western blot (WB) analysis in control and T1D rat testes. The histogram shows the F-actin/G-actin optical density (OD) ratio. (**B**) Immunofluorescence (IF) analysis of F-ACT (red) in the testes of control and T1D animals. Slides were counterstained with 4′,6-diamidino-2-phenylindole (DAPI) fluorescent nuclear staining (blue). The scale bars represent 20 μm and 10 μm in the insets. Dotted arrows: spermatids (SPT); arrowheads: Sertoli cells (SC). (**C**) Quantification of F-ACT fluorescence signal intensity normalized to DAPI signal. Data are expressed as mean ± standard error of the mean (SEM) from five animals in each group. **: *p* < 0.01. Each experiment was performed in triplicate.

Figure 1 shows the effects of T1D on testicular actin organization.

Biochemical fractionation of G- and F-actin followed by Western blot (WB) analysis revealed a significant reduction in the F-actin/G-actin ratio in T1D animals compared with controls (*p* < 0.01; Figure 1A), indicating an altered F-/G-actin homeostasis. To further investigate the spatial organization of the actin cytoskeleton, immunofluorescence (IF) analysis was performed (Figure 1B). In control testis, F-actin (red) displayed a continuous and highly organized distribution, clearly identifiable at the basal compartment in proximity to the BTB, consistent with preserved cytoskeletal architecture (arrowhead, inset). In addition, F-actin signal exhibited a characteristic striped-like arrangement at the interface between the SC cytoplasm and the heads of elongating SPT, reflecting the organized actin bundles associated with the ES (arrow). Conversely, testes from T1D animals showed marked F-actin disorganization, characterized by a fragmented, diffuse-staining pattern, suggesting structural alterations of the actin cytoskeleton. In particular, the signal appeared irregular and discontinuous in regions corresponding to the BTB (arrowhead) and SC cytoplasmic protrusions surrounding SPT heads (arrow). Finally, quantitative analysis of F-actin fluorescence intensity (Figure 1C) confirmed a significant reduction in T1D animals compared with controls (*p* < 0.01), in agreement with the reduced F-actin/G-actin ratio.

### 2.2. Effects of T1D on ABPs

**Figure 2:**
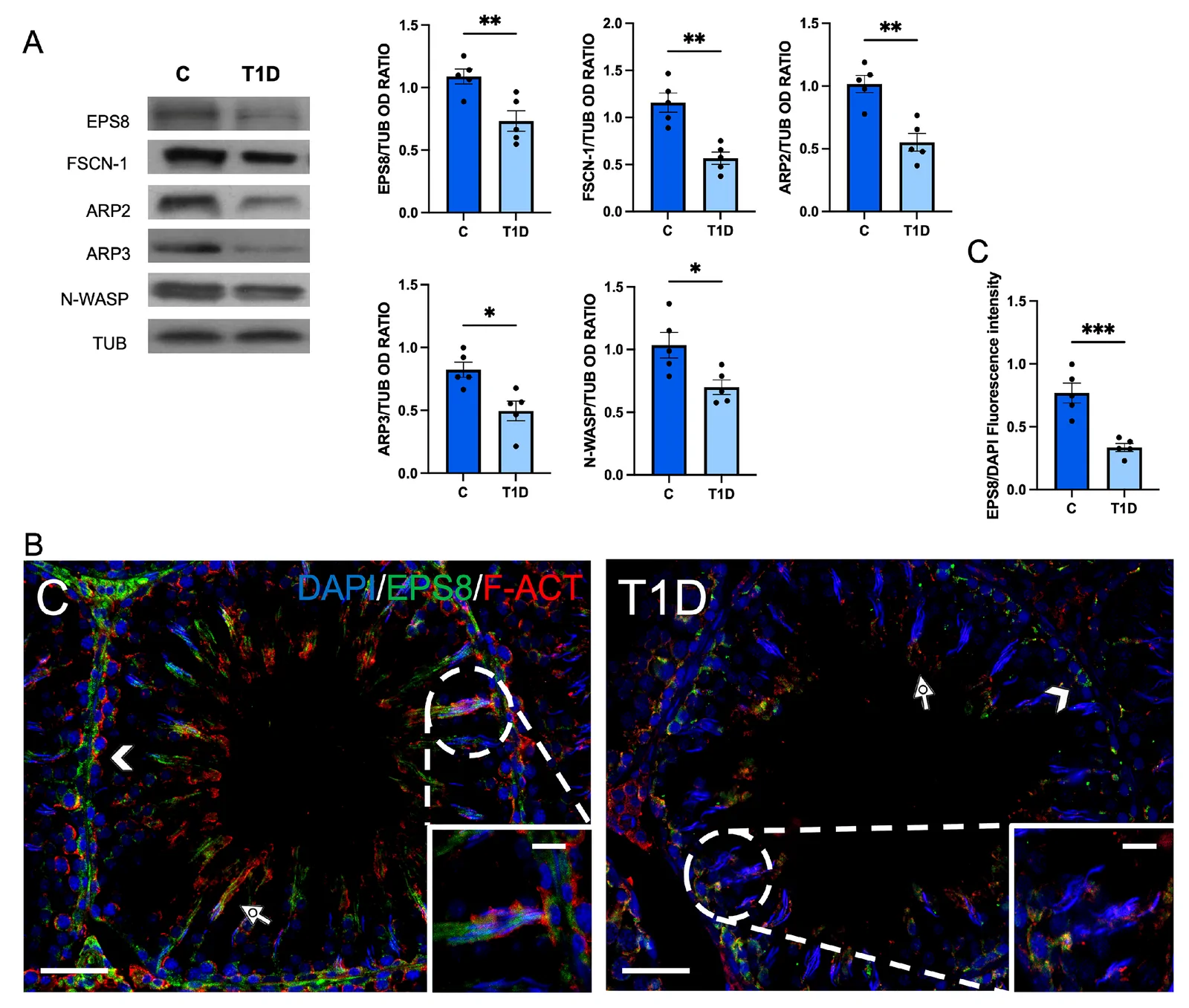
Analysis of the levels and localization of actin-binding proteins (ABPs) in control and T1D rat testes. (**A**) WB analysis of Epidermal Growth Factor Receptor Kinase Substrate 8 (EPS8), Fascin-1 (FSCN-1), Actin-Related Proteins 2 and 3 (Arp2 and Arp3), and Neuronal Wiskott-Aldrich Syndrome Protein (N-WASP) in control and T1D rat testes. The histograms show their relative protein level. Data were normalized to α-tubulin (TUB) and reported as an OD ratio. (**B**) IF analysis of EPS8 (green) and F-ACT (red) in the testes of control and T1D animals. Slides were counterstained with DAPI fluorescent nuclear staining (blue). The scale bars represent 20 μm and 10 μm in the insets. Dotted arrows: SPT; arrowheads: SC. (**C**) Histogram showing the quantification of EPS8 fluorescence intensity normalized to the DAPI signal. Data are expressed as mean ± SEM from five animals in each group. *: *p* < 0.05; **: *p* < 0.01; ***: *p* < 0.001. Each experiment was performed in triplicate.

To further investigate the molecular mechanisms underlying actin cytoskeleton remodeling, the protein levels of key ABPs were evaluated by WB analysis (Figure 2A).

A significant reduction in EPS8 levels was observed in T1D samples compared with controls (*p* < 0.01). Similarly, FSCN-1 protein levels were markedly decreased in T1D animals (*p* < 0.01). Components of the Arp2/3 complex were also significantly affected, as both ARP2 (*p* < 0.01) and ARP3 (*p* < 0.05) protein levels were reduced in diabetic testes. In addition, N-WASP protein levels were significantly reduced in the T1D group (*p* < 0.05). Overall, these findings indicate that T1D broadly affects proteins involved in actin filament bundling, stabilization, and branching.

IF analysis was subsequently performed to evaluate the localization of EPS8 in relation to F-actin organization (Figure 2B). In control testes, EPS8 (green) showed strong overlap with F-actin (red) along the SE, displaying a well-organized and continuous pattern, particularly in regions corresponding to cell–cell junctions (arrowhead) and ES (dotted arrow, insets). In contrast, T1D samples exhibited a marked reduction in EPS8 signal together with reduced overlap with the F-actin. The staining pattern appeared fragmented and diffuse, especially within the basal compartment of the seminiferous tubules.

Quantitative analysis of EPS8 fluorescence intensity (Figure 2C) confirmed a reduction in T1D animals compared with controls (*p* < 0.001), consistent with the WB results.

### 2.3. Effects of T1D on Wnt/PCP Signaling Components

**Figure 3:**
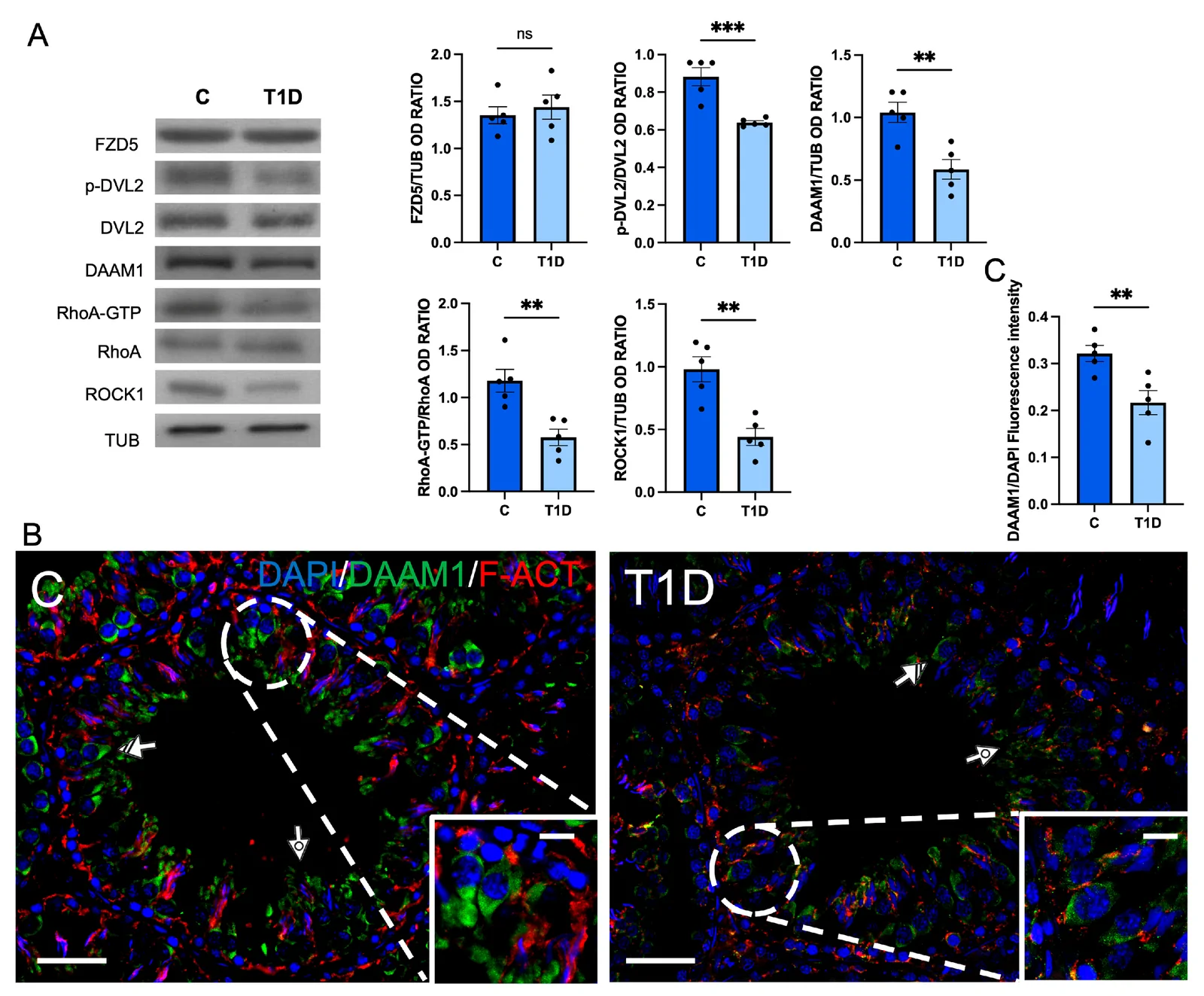
Analysis of the levels and localization of Planar Cell Polarity (PCP) pathway components in control and T1D rat testes. (**A**) WB analysis of Frizzled-5 (FZD5), phospho-Disheveled 2 (p-DVL2), Disheveled-2 (DVL), Disheveled-Associated Activator of Morphogenesis 1 (DAAM1), GTP-bound Ras Homolog Family Member A (RhoA-GTP), Ras Homolog Family Member A (RhoA), Rho-Associated Protein Kinase 1 (ROCK1) in control and T1D rat testes. The histograms show the relative protein or phosphorylation levels. Data were normalized to TUB or to the corresponding total protein levels and reported as an OD ratio. (**B**) IF analysis of DAAM1 (green) and F-ACT (red) in the testes of control and T1D animals. Slides were counterstained with DAPI fluorescent nuclear staining (blue). The scale bars represent 20 μm and 10 μm in the insets. Striped arrows: spermatocytes (SPC); Dotted arrows: SC. (**C**) Histogram showing the quantification of DAAM1 fluorescence intensity normalized to the DAPI signal. Data are expressed as mean ± SEM from five animals in each group. ns: not significant; **: *p* < 0.01; ***: *p* < 0.001. Each experiment was performed in triplicate.

Given the alterations in actin cytoskeleton organization and ABPs, we next investigated the involvement of the non-canonical Wnt/PCP signaling pathway (Figure 3).

Figure 3A shows that WB analysis revealed no significant differences in FZD5 expression between control and T1D groups (ns). In contrast, phosphorylated DVL2 was markedly reduced in T1D testes compared with controls (*p* < 0.001). Consistently, DAAM1 protein levels were significantly reduced in diabetic animals (*p* < 0.01). Downstream signaling components were also affected. The active form RhoA-GTP was significantly reduced in T1D testes, resulting in a lower RhoA-GTP/RhoA ratio (*p* < 0.01). In addition, ROCK1 expression was significantly downregulated (*p* < 0.01). Collectively, these findings indicate dysregulation of the PCP signaling pathway and its downstream effectors involved in actin cytoskeleton remodeling.

IF analysis further supported these observations (Figure 3B). In control testes, DAAM1 (green) exhibited a defined localization pattern along the SE, showing a predominant localization in spermatocytes (SPC; striped arrows and insets). Only limited overlap with F-actin (red) was observed, mainly at the ES (dotted arrow). In contrast, T1D samples exhibited markedly reduced DAAM1 immunoreactivity, associated with a disorganized and diffuse staining pattern compared with controls. The residual signal appeared weaker and less structured throughout the SE. The analysis of DAAM1 fluorescence intensity (Figure 3C) confirmed a significant decrease in T1D animals compared with controls (*p* < 0.01), consistent with the WB results.

### 2.4. Effects of T1D on the LIMK/Cofilin Pathway

**Figure 4:**
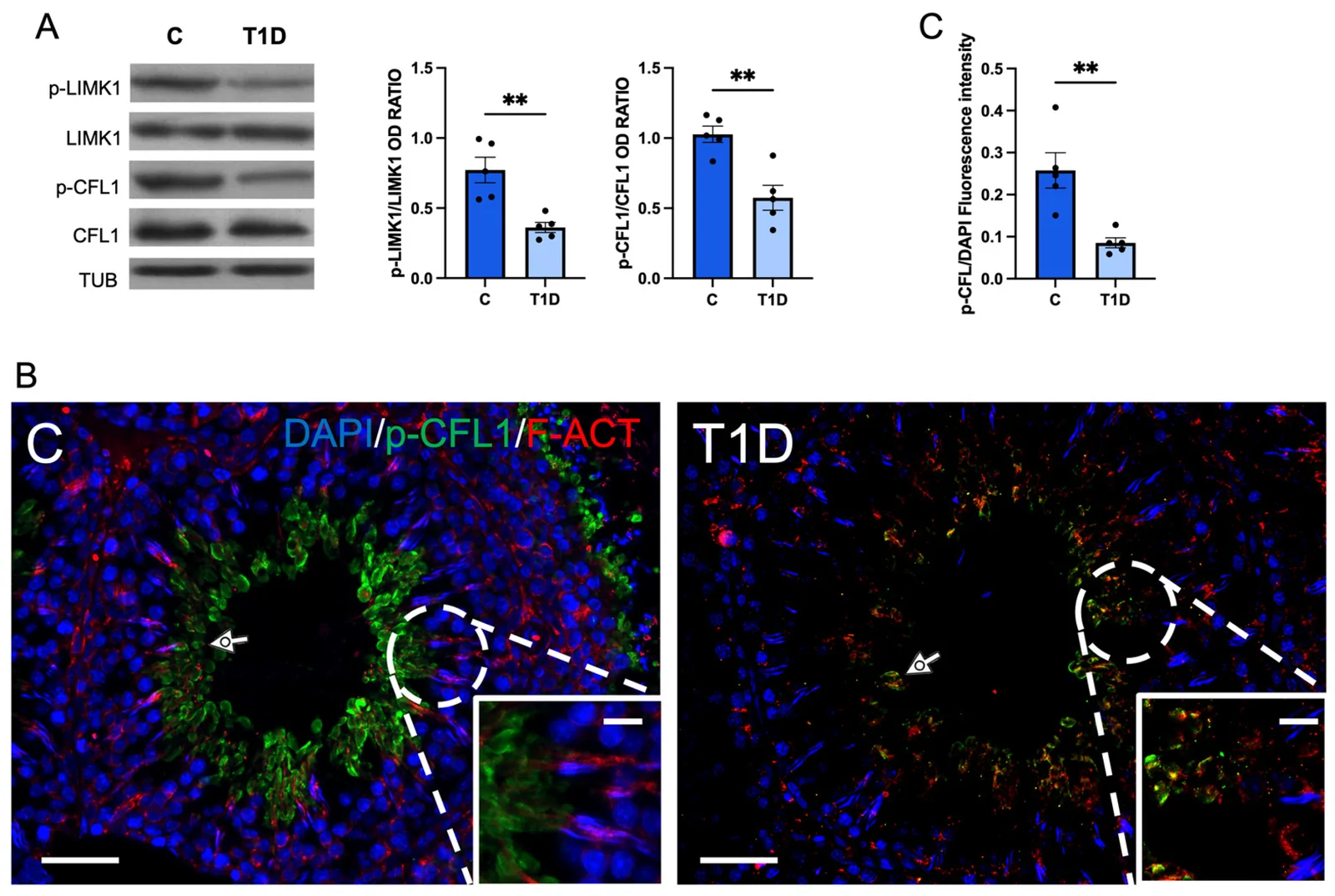
Analysis of the levels and localization of LIM Domain Kinase 1 (LIMK1) and Cofilin 1 (CFL1) in control and T1D rat testes. (**A**) WB analysis of phospho-LIMK1 (p-LIMK1), LIMK1, phospho-CFL1 (p-CFL1), and CFL1 in control and T1D rat testes. The histograms show the relative phosphorylation levels; data were normalized to the corresponding total protein levels and reported as an OD ratio. (**B**) IF analysis of p-CFL1 (green) and F-ACT (red) in the testes of control and T1D animals. Slides were counterstained with DAPI fluorescent nuclear staining (blue). The scale bars represent 20 μm and 10 μm in the insets. Dotted arrows: SPT. (**C**) Histogram showing the quantification of p-CFL1 fluorescence intensity normalized to the DAPI signal. Data are expressed as mean ± SEM from five animals in each group. **: *p* < 0.01. Each experiment was performed in triplicate.

To further investigate the downstream signaling events linking RhoA/ROCK1 activity to actin cytoskeleton remodeling, the LIMK/cofilin pathway was analyzed (Figure 4).

WB analysis revealed significantly reduced phosphorylated LIMK1 (p-LIMK1) levels in T1D samples compared with controls (*p* < 0.01). Similarly, phosphorylated cofilin (p-CFL1) levels were significantly decreased in diabetic animals (*p* < 0.01).

IF analysis further supported these observations (Figure 4B). In control testes, p-CFL1 (green) exhibited a strong and well-defined distribution within the SE, with prominent localization in the adluminal compartment, particularly along the cytoplasmic regions surrounding elongating SPT (dotted arrows and insets). In contrast, T1D samples displayed markedly reduced p-CFL1 immunoreactivity, associated with a weaker, fragmented, and less organized staining pattern, particularly within the adluminal compartment.

Quantitative fluorescence analysis (Figure 4C) confirmed a significant reduction in p-CFL1 fluorescence intensity in T1D animals compared with controls (*p* < 0.01), consistent with the WB results.

### 2.5. Effects of T1D on the RICTOR/PKC/MARCKS Pathway

**Figure 5:**
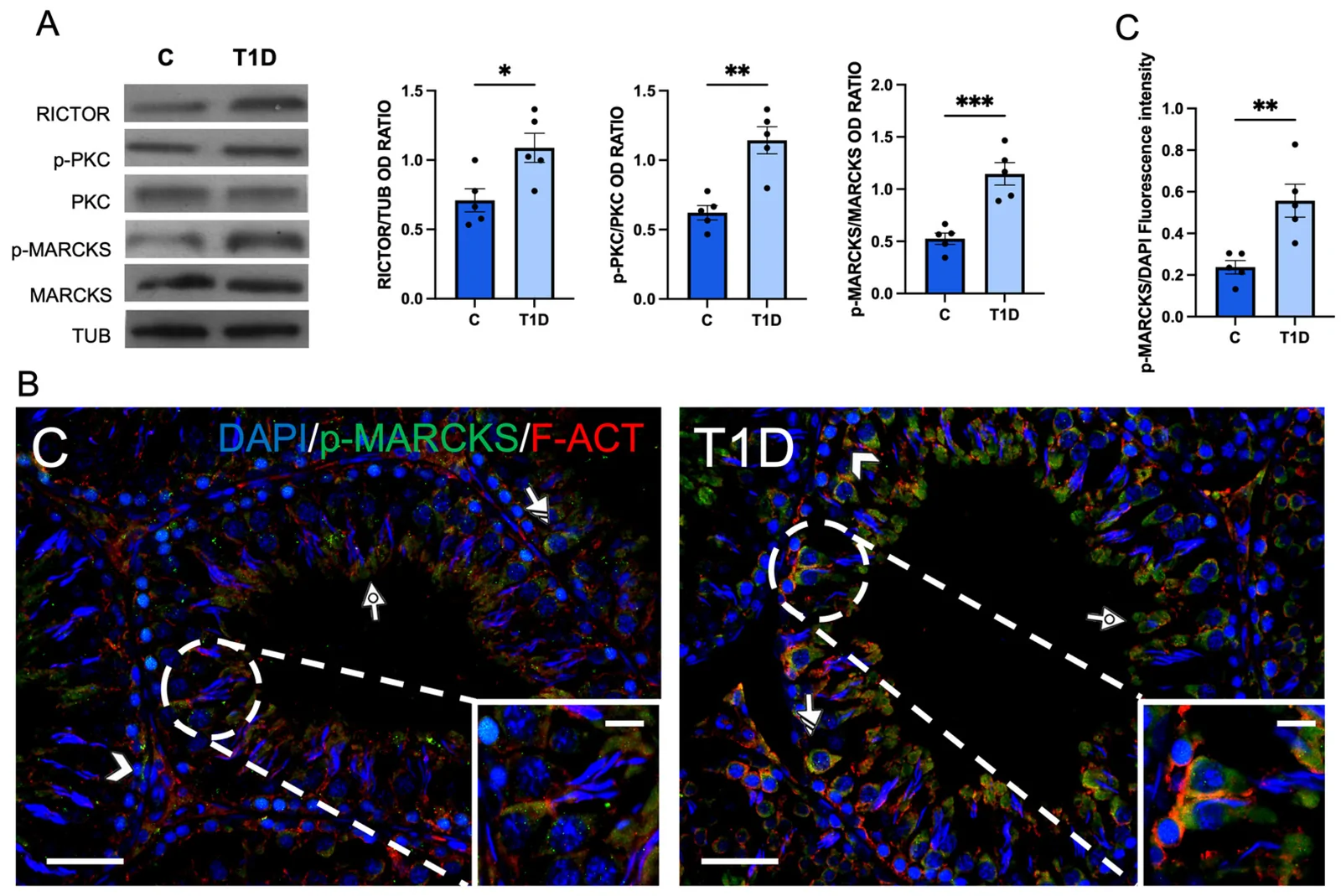
Analysis of the levels and localization of Protein Kinase C (PKC) pathway components in control and T1D rat testes. (**A**) WB analysis of Rapamycin-Insensitive Companion of Mammalian Target of Rapamycin (RICTOR), phospho-PKC (p-PKC), PKC, phospho-Myristoylated Alanine-Rich C-Kinase Substrate (p-MARCKS), and MARCKS in control and T1D rat testes. The histograms show the relative protein or phosphorylation levels. Data were normalized to TUB or to the corresponding total protein levels and reported as an OD ratio. (**B**) IF analysis of p-MARCKS (green) and F-ACT (red) in the testes of control and T1D animals. Slides were counterstained with DAPI fluorescent nuclear staining (blue). The scale bars represent 20 μm and 10 μm in the insets. Striped arrows: SPC; dotted arrows: SPT; arrowheads: SC. (**C**) Histogram showing the quantification of p-MARCKS fluorescence signal normalized to the DAPI signal. Data are expressed as mean ± SEM from five animals in each group. *: *p* < 0.05; **: *p* < 0.01; ***: *p* < 0.001. Each experiment was performed in triplicate.

To further investigate additional signaling pathways potentially involved in diabetes-induced cytoskeletal alterations, the RICTOR/PKC/MARCKS axis was examined (Figure 5).

WB analysis revealed a significant increase in RICTOR protein levels in T1D testes compared with controls (*p* < 0.05). Consistently, phosphorylated PKC (p-PKC; *p* < 0.01) and phosphorylated MARCKS (p-MARCKS; *p* < 0.001) levels were also increased in diabetic animals.

IF analysis further supported these observations (Figure 5B). In control testes, p-MARCKS (green) displayed a well-defined distribution pattern within the SE, with prominent localization along the cytoplasmic regions of SPC (striped arrows) and elongating SPT facing the tubular lumen (dotted arrows and insets), exhibiting a partial spatial overlap with F-actin (red). In T1D samples, p-MARCKS immunoreactivity appeared markedly increased and more diffusely distributed throughout the adluminal compartment, with loss of the organized pattern observed in control tubules. In particular, enhanced p-MARCKS staining was evident within the adluminal compartment, especially in regions corresponding to SPC and the apical cytoplasmic regions surrounding elongating SPT.

Quantitative fluorescence analysis (Figure 5C) confirmed a significant increase in p-MARCKS fluorescence intensity in T1D testes compared with controls (*p* < 0.01), consistent with the WB results.

### 2.6. Effects of T1D on Sperm Actin Cytoskeleton Organization

**Figure 6:**
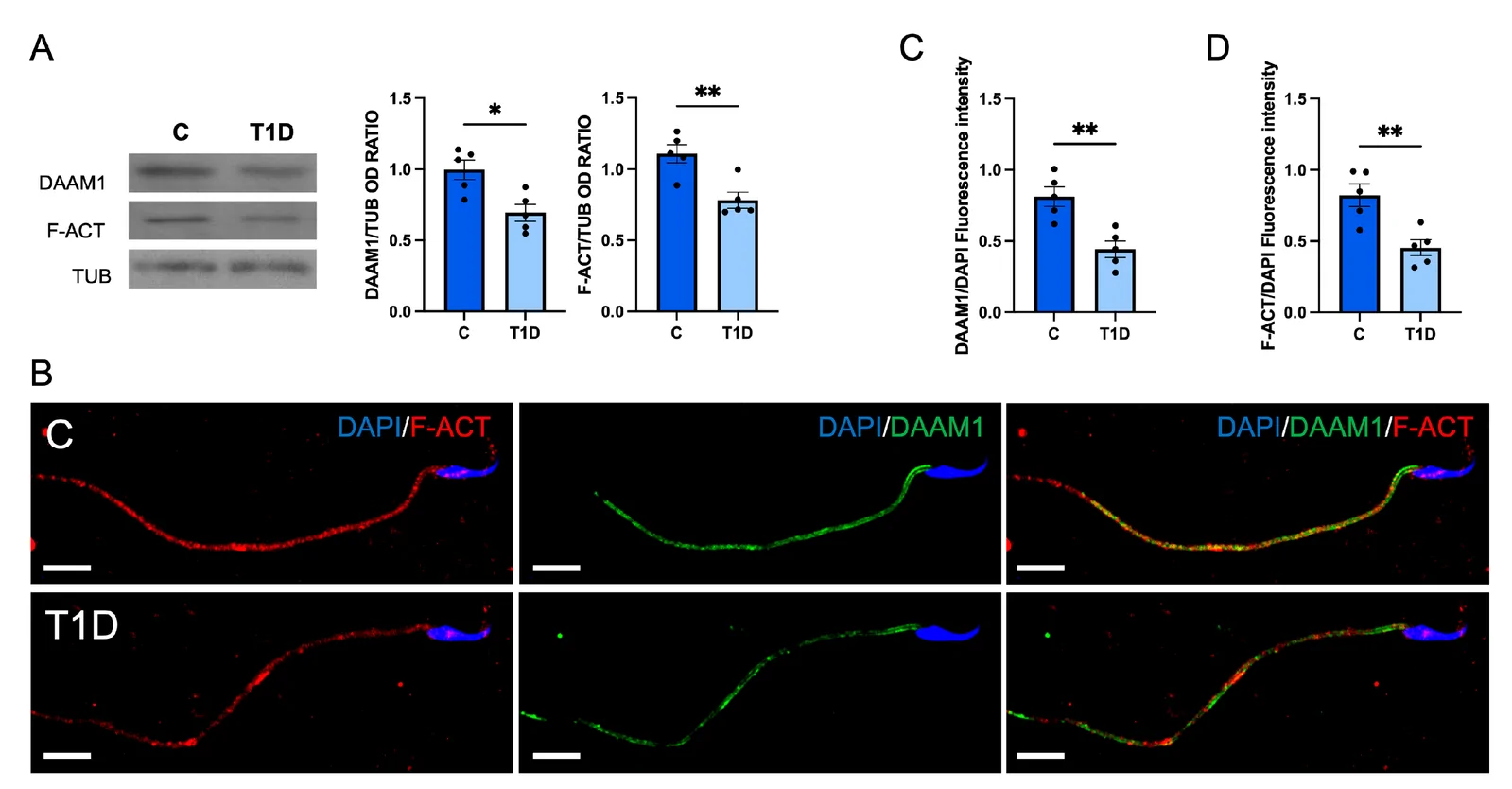
Analysis of the levels and localization of DAAM1 and F-ACT in control and T1D rat spermatozoa (SPZ). (**A**) WB analysis of DAAM1 and F-ACT in control and T1D rat SPZ. The histograms show their relative protein levels. Data were normalized to TUB and reported as OD ratios. (**B**) IF analysis of DAAM1 (green) and F-ACT (red) in the SPZ of control and T1D animals. Slides were counterstained with DAPI fluorescent nuclear staining (blue). The scale bars represent 20 μm. (**C**,**D**) Histograms showing the quantification of DAAM1 (**C**) and F-ACT (**D**) fluorescence intensity normalized to the DAPI signal. Data are expressed as mean ± SEM from five animals in each group. *: *p* < 0.05; **: *p* < 0.01. Each experiment was performed in triplicate.

To determine whether the diabetes-induced cytoskeletal alterations identified in the testis persist in the mature male gamete, the expression of DAAM1 and F-actin was evaluated by WB and IF analyses. (Figure 6).

WB analysis revealed a significant reduction in DAAM1 protein levels in SPZ from T1D animals compared with controls (*p* < 0.05; Figure 6A). Consistently, F-actin levels were also markedly decreased in the T1D group (*p* < 0.01).

IF analysis further supported these findings (Figure 6B). In control SPZ, DAAM1 and F-actin showed a well-organized distribution pattern along the sperm flagellum, with extensive overlap in the merged images. In T1D SPZ, both DAAM1 and F-actin fluorescence signals were markedly reduced, resulting in weaker overlap. Quantitative fluorescence analysis (Figure 6C,D) confirmed a significant reduction in both DAAM1 and F-actin fluorescence intensity in T1D SPZ compared with controls (*p* < 0.01 for both), consistent with the WB results.

### 2.7. Effects of T1D on AR and p-MARCKS Activation in SPZ

**Figure 7:**
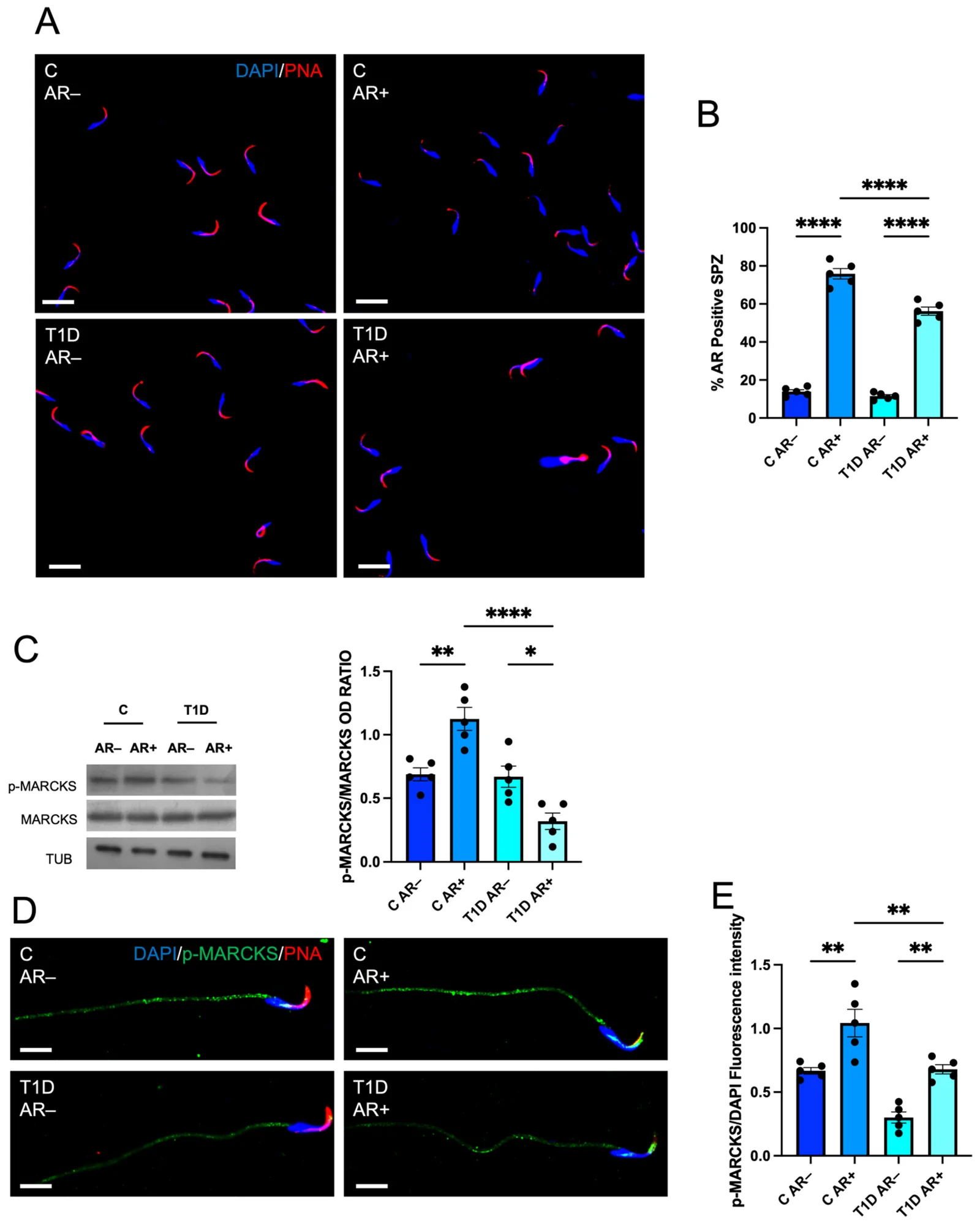
Analysis of acrosome reaction (AR) and p-MARCKS levels in control and T1D rat SPZ. (**A**) Representative fluorescence images of acrosome-reacted (AR+) and non-reacted (AR−) SPZ stained with Peanut Agglutinin (PNA; red) and DAPI (blue). (**B**) Quantitative analysis of the percentage of AR+ SPZ in control and T1D rat SPZ. (**C**) WB analysis of p-MARCKS and MARCKS in AR− and AR+ SPZ from control and T1D rats. The histogram shows the relative p-MARCKS phosphorylation levels. Data were normalized to the corresponding total MARCKS levels and reported as an OD ratio. (**D**) IF analysis of p-MARCKS (green) and PNA (red) in the experimental groups. Slides were counterstained with DAPI fluorescent nuclear staining (blue). The scale bars represent 20 μm. (**E**) Histogram showing the quantification of p-MARCKS fluorescence intensity normalized to the DAPI signal. Data are expressed as mean ± SEM from five animals in each group. *: *p* < 0.05; **: *p* < 0.01; ****: *p* < 0.0001. Each experiment was performed in triplicate.

To evaluate the ability of SPZ from control and T1D animals to undergo a proper AR, sperm samples were incubated in vitro with progesterone and subsequently stained with Peanut Agglutinin (PNA; Figure 7A).

IF analysis allowed discrimination between acrosome-reacted (AR+) and non-reacted (AR−) SPZ based on the PNA fluorescence pattern in the acrosomal region (red), with AR– SPZ exhibiting a clear acrosomal fluorescent signal and AR+ SPZ showing markedly reduced or absent PNA staining. Quantitative analysis confirmed that progesterone treatment efficiently induced the AR in control samples, resulting in a significantly higher percentage of AR+ SPZ compared with the AR− condition (*p* < 0.0001; Figure 7B). In contrast, T1D SPZ exhibited a significantly reduced ability to undergo progesterone-induced AR compared with controls, showing an approximately 26% reduction in the percentage of AR+ cells (*p* < 0.001). Despite this decrease, the percentage of AR+ SPZ in T1D animals remained significantly higher than in the respective AR− group (*p* < 0.001). No significant differences were observed between control AR− and T1D AR− samples.

To investigate the molecular mechanisms associated with the impaired acrosomal response observed in T1D SPZ, p-MARCKS levels were evaluated by WB analysis (Figure 7C). Results revealed a significant increase in the p-MARCKS/MARCKS ratio in control AR+ SPZ compared with the corresponding AR− condition (*p* < 0.01). In contrast, T1D AR+ SPZ exhibited a significantly lower p-MARCKS/MARCKS ratio compared with control AR+ SPZ (*p* < 0.0001). Moreover, although p-MARCKS levels were significantly increased in T1D AR+ compared with the corresponding AR− condition (*p* < 0.05), they remained markedly lower than those observed in control AR+ SPZ.

IF analysis supported these findings (Figure 7D). In control AR+ SPZ, p-MARCKS immunoreactivity appeared more intense, particularly in the acrosomal region and along the flagellum, whereas T1D AR+ SPZ exhibited a weaker fluorescence signal. Quantitative fluorescence analysis (Figure 7E) confirmed the reduced p-MARCKS fluorescence intensity in T1D AR+ SPZ compared with control AR+ SPZ (*p* < 0.01), consistent with the WB results.

## 3. Discussion

In recent decades, the global prevalence of T1D has gradually increased and, although it has traditionally been considered a disease primarily affecting children, epidemiological evidence indicates that new diagnoses in adults now exceed those in younger individuals [54]. Chronic T1D-associated hyperglycemia is a major driver of the activation of aberrant metabolic pathways, promoting excessive ROS production and impairment of cellular antioxidant mechanisms, including in the testis [50,51]. These phenomena support the establishment of a persistent OS state that may cause structural and functional damage to cell organelles and macromolecules. The cytoskeleton is among the major components susceptible to oxidative damage, with its dynamic organization being particularly affected, as documented by several reports [55,56,57,58]. In this regard, in our previous studies using the same T1D rat model, we demonstrated altered redox homeostasis, BTB disruption, microtubule disorganization, impaired GC proliferation and meiotic progression, and decreased sperm quality, including reduced sperm count, viability, and motility [52,53]. Building upon this biological framework, the present study identifies the actin cytoskeleton as an additional target of diabetic damage. Although OS was not directly investigated, our previous findings, together with the available literature, support the hypothesis that it may represent a plausible upstream mechanism underlying the actin cytoskeletal alterations observed here.

Actin microfilaments are essential to support spermatogenesis; indeed, within the SE, highly organized actin filament networks regulate SC–GC adhesion, epithelial polarity, BTB dynamics, SPT transport, and spermiation. Disruption of this extremely organized network may profoundly affect GC differentiation and, therefore, male fertility. Our WB and IF analyses revealed reduced F-actin abundance and an altered distribution in T1D testes, together with a decreased F-actin/G-actin ratio, indicating a disruption of actin cytoskeleton organization. These results align with the well-known susceptibility of the actin cytoskeleton to oxidative and metabolic stress, which impairs cellular architecture and function [59,60,61,62,63].

The altered F-actin organization was accompanied by decreased protein levels of several ABPs involved in actin filament stabilization, nucleation, and remodeling. EPS8 and FSCN-1 are key actin-bundling proteins promoting the formation of packed actin filament arrays, typical of the ES. The reduction in the levels of both proteins is consistent with impaired actin filament bundling at SC-SC and SC-GC interfaces [16,17,19,64]. Accordingly, previous studies have shown that disruption of EPS8 or FSCN-1 leads to defective SPT adhesion and abnormal spermiation, supporting the hypothesis that analogous mechanisms may underlie the testicular alterations observed in T1D animals.

The dynamic organization of actin microfilaments within the SE also relies on a tightly regulated balance between bundled and branched actin networks, controlled by several factors, including N-WASP and the Arp2/3 complex [65,66,67]. Worth remembering, bundled filaments sustain the stability of adhesion complexes, while branched networks preserve the required plasticity for GC movement [68]. Interestingly, the concomitant reduction in N-WASP, Arp2, and Arp3 protein levels, together with the altered F-actin/G-actin ratio, is consistent with a disruption of the physiological balance between actin filament polymerization and depolymerization, ultimately affecting actin cytoskeleton remodeling rather than selectively altering a single component of this process.

The impairment of factors involved in actin dynamics suggests that T1D may affect upstream signaling networks controlling cytoskeletal remodeling. In this regard, the PCP pathway is of particular interest, given its well-established role in coordinating cell polarity and actin organization within the SE [20]. Consistent with this hypothesis, testes of T1D animals exhibited reduced DVL2 phosphorylation, decreased DAAM1 and ROCK1 expression, and diminished RhoA activation. Interestingly, the unaffected FZD5 expression suggests an impairment of intracellular PCP signaling activation rather than receptor availability. Consistently, T1D testes also showed reduced phosphorylation of LIMK1 and, in turn, of CFL1. Since LIMK1-mediated phosphorylation inhibits CFL1 activity [69], the concurrent decrease in p-LIMK1 and p-CFL1 is consistent with altered regulation of CFL1 activity, potentially favoring increased actin filament severing and destabilization. These findings are in line with the marked F-actin disorganization observed in diabetic testes and further support the concept that T1D may disrupt actin remodeling pathways within the SE. Interestingly, under physiological conditions, p-CFL1 exhibited a prominent localization within the adluminal compartment of the SE, particularly surrounding elongating SPT, which encompasses the apical ES, one of the most actin-rich and dynamic junctional structures of the SE. This spatial distribution is consistent with the tightly regulated spatiotemporal control of CFL1 activity required for actin cytoskeleton remodeling at the SC–SPT interface during the final stages of SPT maturation. Indeed, CFL1 is a key regulator of actin filament turnover and has been identified as a component of tubulobulbar complexes, actin-rich structures involved in the remodeling of the apical ES preceding spermiation [70,71,72]. In T1D testes, the marked reduction and altered distribution of p-CFL1 immunoreactivity within the adluminal compartment suggest that the spatial regulation of the LIMK1/CFL1 axis is disrupted, potentially impairing actin remodeling at the apical ES. This interpretation is further supported by our previous findings in the same experimental model, which demonstrated a significant reduction in sperm number [53], suggesting that dysregulated actin remodeling at the SC–SPT interface may represent one of the mechanisms contributing to the reduced sperm output observed in T1D.

In contrast to the suppression of PCP signaling, the mTORC2 pathway showed an opposite trend. Previous reports have demonstrated altered PKC regulation in different systems of diabetic models [73], including the testis [48,74]. In the present study, we confirmed increased RICTOR expression and enhanced phosphorylation of PKC and, to the best of our knowledge, report for the first time increased MARCKS phosphorylation in diabetic testes. It is well established that mTORC2 regulates cytoskeletal organization through PKC-dependent signaling [37], whereas MARCKS acts as a membrane-associated ABP that stabilizes cortical actin networks by cross-linking F-actin [38,75]. Upon PKC-mediated phosphorylation, MARCKS dissociates from the plasma membrane, resulting in the loss of its actin cross-linking activity and promoting cytoskeletal remodeling. Therefore, dysregulation of the PKC/MARCKS axis may further contribute to altered F-actin organization observed in diabetic testes, acting in concert with the PCP/LIMK1/CFL1 pathway to disrupt actin cytoskeleton homeostasis. Given that proper F-actin architecture is essential for maintaining BTB integrity and supporting GC development, these alterations may represent a mechanistic link between diabetes-induced signaling abnormalities, BTB disruption, and impaired spermatogenesis.

Notably, our previous study using the same experimental model demonstrated that T1D is associated with significant impairments in sperm quality and function, including alterations of the sperm microtubule cytoskeleton [53]. The present study extends these observations by showing that actin cytoskeletal alterations are also evident in mature SPZ, as demonstrated by the reduced expression of both DAAM1 and F-actin. These findings indicate that diabetes-induced cytoskeletal dysregulation is not restricted to the SE but extends to mature SPZ, suggesting that defects in actin remodeling acquired during spermatogenesis may persist throughout sperm maturation. This interpretation is consistent with previous studies showing that metabolic disturbances, including obesity, can induce long-lasting molecular alterations in mature SPZ, extending the impact of systemic disease beyond the testicular environment and into the post-testicular phases of sperm maturation [76,77]. Since actin remodeling is essential for sperm motility, capacitation, and acrosomal function [14,78,79,80], persistent disruption of the actin cytoskeleton may contribute to the functional impairment of diabetic SPZ. Together with our previous findings on microtubule alterations [53], the present results suggest that T1D induces broad cytoskeletal dysregulation affecting both actin and microtubule networks, thereby compromising SE homeostasis and mature sperm function.

Among the numerous events required for successful fertilization, the AR represents one of the most critical functional processes. This event relies on tightly regulated actin remodeling, characterized by transient actin polymerization followed by depolymerization, which enables the fusion of the outer acrosome membrane and the plasma membrane, and the subsequent release of hydrolytic enzymes required for zona pellucida penetration [78]. In the present study, T1D SPZ exhibited a reduced percentage of AR+ cells, indicating impaired acrosomal responsiveness. Interestingly, AR+ SPZ from control animals displayed increased p-MARCKS levels, supporting its physiological involvement in cytoskeletal remodeling that accompanies AR [81]. Conversely, despite the increased p-MARCKS levels observed in the diabetic testes, T1D SPZ failed to exhibit the physiological increase in p-MARCKS during AR. This apparent discrepancy suggests that T1D may not simply alter the extent of MARCKS phosphorylation but rather disrupt its spatial and temporal regulation. Consequently, diabetes-associated dysregulation of MARCKS signaling may impair the coordinated actin cytoskeletal remodeling required for an efficient AR, consistent with the reduced percentage of AR+ SPZ observed in T1D animals. Given the essential role of the AR in successful fertilization, the reduced acrosome responsiveness observed in T1D SPZ is likely to have functional consequences. Together with the decreased p-MARCKS expression, these results support the hypothesis that diabetes-induced cytoskeletal dysregulation compromises sperm fertilizing competence. Therefore, these data are indicative of impaired sperm function and reduced fertilizing potential. Although our findings strongly support a primary testicular origin of the observed sperm abnormalities, epididymal function was not specifically investigated. Thus, a contribution of post-testicular events cannot be completely excluded and warrants further investigation.

Some limitations of the present study should be acknowledged. Although our findings demonstrate significant alterations in signaling pathways associated with cytoskeletal remodeling and sperm function, direct fertility assays were not performed. In addition, while conventional fluorescence microscopy allowed the overall distribution of F-actin within the SE to be evaluated, confocal microscopy with z-stack acquisition would provide a more detailed three-dimensional assessment of actin organization within the SC. Furthermore, although the observed changes in the PCP/LIMK1/CFL1 and mTORC2/PKC/MARCKS signaling pathways were closely associated with actin cytoskeleton disorganization and impaired AR, a direct causal relationship was not experimentally demonstrated. Future mechanistic studies employing pharmacological or genetic modulation of these pathways, together with rescue approaches, will be necessary to define their specific contribution to T1D-induced alterations in actin cytoskeleton remodeling and the consequent testicular and sperm dysfunction.

## 4. Materials and Methods

### 4.1. Animals, Treatments, and Sample Collection

Ten adult male Wistar rats (*Rattus norvegicus*), aged two months and weighing 220 ± 18.97 g, were individually housed in stainless-steel cages under controlled environmental conditions (12 h light/dark cycle, temperature of 24 ± 2 °C, and relative humidity of 55 ± 20%). To ensure adequate adaptation to the new environment, the animals underwent a one-week acclimatization period before the experiments began. Throughout the study, the animals had free access to sterile food and water. The rats were randomly divided into two groups: a control group (C; *n* = 5), which received 5% citrate buffer solution (#211018; AppliChem GmbH, Darmstadt, Germany), and the treated group (T1D; *n* = 5), which received a single intraperitoneal injection of streptozotocin (65 mg/kg body weight) (#18883-66-4; Chem Cruz Biochemicals; Huissen, The Netherlands) dissolved in the same buffer [52,53]. Blood glucose levels were regularly monitored throughout the experimental period, as described in our previous study [52], confirming persistent hyperglycemia in T1D animals. After three months, the animals were anesthetized with an intraperitoneal injection of chloral hydrate (#15307; Sigma-Aldrich; Milan, Italy) followed by the administration of a lethal dose of urethane (2 g/kg; #94300; Sigma-Aldrich; Milan, Italy). Immediately after euthanasia, testes were excised and rinsed in pre-warmed phosphate-buffered saline (PBS; P3813; Sigma-Aldrich; Milan, Italy). The left testes were fixed in Bouin’s solution (#HT10132; Sigma-Aldrich; Milan, Italy) for histological analysis, whereas the right testes were snap-frozen in liquid nitrogen and stored at −80 °C for molecular and biochemical analyses. SPZ were collected by mincing the epididymides in PBS, followed by filtration and microscopic inspection to exclude somatic cell contamination. An aliquot of freshly isolated SPZ was processed immediately for in vitro AR experiments (see Section 4.2). Additional aliquots were air-dried on microscope slides and stored at −20 °C for staining analyses, while the remaining samples were centrifuged at 1000× *g* for 15 min at 4 °C and stored at −80 °C for downstream molecular analyses. All experimental procedures were approved by the Animal Ethics Committee of the University of Campania “Luigi Vanvitelli” of Naples and by the Italian Ministry of Health (protocol no. 30/2021, approval date: 10 November 2021). Animal care complied with Italian Legislative Decree 26/2014, implementing Directive 2010/63/EU on the protection of animals used for scientific purposes. All efforts were made to reduce both the number of animals used and their suffering during the study.

### 4.2. In Vitro AR Induction

To induce AR, SPZ from C and T1D animals were incubated with 15 μM progesterone dissolved in ethanol at 37 °C for 2 h. At the end of the incubation period, aliquots of the sperm suspension were spotted onto microscope slides, air-dried, and stored at −20 °C for subsequent staining analyses. The remaining samples were centrifuged, the supernatant was discarded, and the resulting pellets were stored at −80 °C for downstream molecular analyses [82].

### 4.3. Protein Extraction and WB Analysis

Total proteins were extracted from testes, freshly isolated SPZ, and progesterone-treated SPZ subjected to in vitro AR by homogenizing the samples in ice-cold RIPA lysis buffer (#TCL131; HiMedia Laboratories GmbH, Einhausen, Germany) supplemented with 10 μL/mL protease (#P8340; Sigma-Aldrich; Milan, Italy) and phosphatase (#P5726; Sigma-Aldrich; Milan, Italy) inhibitor cocktails. Homogenates were centrifuged at 14,000× *g* for 30 min at 4 °C, and the resulting supernatants were collected and stored at −80 °C until use. Protein concentrations were determined using the colorimetric Lowry assay [83].

Active RhoA levels (RhoA-GTP) were assessed in testicular samples using the Active Rho Detection Kit (#8820, Cell Signaling Technology, Danvers, MA, USA), according to the manufacturer’s instructions. Briefly, GTP-bound RhoA was affinity-purified using glutathione beads coated with the GST-tagged Rho-binding domain (RBD) of Rhotekin and subsequently detected by WB with a RhoA-specific antibody.

For WB analysis, 40 μg of total protein per sample was separated by SDS-PAGE (9–15% polyacrylamide; #A4983; AppliChem GmbH, Darmstadt, Germany) and processed as previously described [84]. Detailed information regarding the primary and secondary antibodies used is reported in Table S1. Protein bands were detected by chemiluminescence and quantified using ImageJ software (version 1.53t; National Institutes of Health, Bethesda, MD, USA). Densitometric values were normalized to α-tubulin. All WB analyses were performed in triplicate.

### 4.4. F-actin/G-actin Fractionation

To assess the F-actin/G-actin balance, G-actin and F-actin fractions were isolated from testicular tissue by differential ultracentrifugation using a protocol by Bhambhvani et al. [85]. Briefly, testes were homogenized in an ice-cold F-actin stabilization buffer containing 100 mM PIPES (pH 6.9), 30% glycerol, 5% dimethyl sulfoxide (DMSO), 1 mM MgSO_4_, 1 mM EGTA, 1% Triton X-100, 1 mM ATP, and a protease inhibitor cocktail. Homogenates were incubated at 37 °C for 10 min and centrifuged at 500× *g* for 5 min to remove tissue debris. The resulting supernatants were ultracentrifuged at 134,000× *g* for 1 h at 37 °C to separate G-actin (supernatant) from F-actin (pellet). The F-actin pellet was resuspended in the same volume as the recovered G-actin fraction using 8 M urea and incubated on ice for 1 h, with vortexing every 15 min to facilitate pellet solubilization. Equal volumes of the corresponding G-actin and F-actin fractions from each sample were then subjected to SDS-PAGE and WB analysis, as described in Section 4.3. Band intensities were quantified by densitometric analysis using ImageJ software, and the F-actin/G-actin ratio was calculated for each sample.

### 4.5. IF Analysis on Testis and SPZ

Testicular tissue sections (5 μm thick) were dewaxed in xylene, rehydrated through a graded ethanol series, and processed as previously described [86]. SPZ were fixed with 4% paraformaldehyde and treated as described in [87]. Slides were incubated with the appropriate primary and secondary antibodies (see Table S1). Negative controls were obtained by omitting the primary antibodies and incubating the sections with the corresponding secondary antibodies. Representative images are shown in Supplementary Figure S1. Slides were mounted with Vectashield + DAPI (#H-1200–10; Vector Laboratories, Peterborough, UK) and coverslipped. Slides were examined under a fluorescence microscope equipped with a UV lamp (Leica DM5000 B + CTR 5000; Leica Microsystems, Wetzlar, Germany), and images were acquired using IM 1000 software (version 4.7.0) and a Leica DFC320 R2 digital camera (Leica Microsystems, Wetzlar, Germany). Quantitative analysis of fluorescence signal intensity and the percentage of positive cells was performed using Fiji (version 3.9.0/ImageJ 1.53t). For each group, 30 tubules/animal (150 tubules total per group) and approximately 300 SPZ per animal were evaluated. All the IF experiments were performed in triplicate.

### 4.6. Statistical Analysis

Statistical analyses were performed using GraphPad Prism software (version 8.0; GraphPad Software, San Diego, CA, USA). Data are expressed as mean ± standard error of the mean (SEM). Comparisons between two groups were performed using the unpaired Student’s *t*-test, whereas comparisons involving more than two groups were analyzed by one-way ANOVA followed by Tukey’s post hoc test. Differences were considered statistically significant at *p* < 0.05.

## 5. Conclusions

This study identifies the actin cytoskeleton as a novel target of T1D-induced reproductive damage. Indeed, T1D was associated with profound alterations in testicular F-actin organization, accompanied by dysregulation of key ABPs and signaling pathways involved in actin cytoskeletal remodeling. In particular, impairment of the PCP signaling pathway and activation of the mTORC2/PKC/MARCKS axis emerged as two complementary mechanisms potentially contributing to actin filament disorganization within the SE, thereby compromising BTB integrity and GC development.

Importantly, these abnormalities were not restricted to the testis but also persisted in mature SPZ, as demonstrated by reduced DAAM1 and F-actin levels, impaired AR, and altered p-MARCKS regulation. The combined data suggest that T1D-induced defects in actin cytoskeleton remodeling originate during spermatogenesis and persist in the mature gamete, providing a mechanistic link between testicular dysfunction and impaired sperm function. Overall, this study expands the current understanding of the molecular mechanisms underlying T1D-associated male reproductive dysfunction and identifies cytoskeletal regulatory pathways as promising targets for future mechanistic studies and therapeutic strategies.

## Data Availability

The original contributions presented in this study are included in the article. Further inquiries can be directed to the corresponding author.

## Supplementary Materials

The following supporting information can be downloaded at: https://www.mdpi.com/article/10.3390/ijms27167423/s1.
