# Supplementary material for: Actin Cytoskeleton Dysregulation Links Testicular and Sperm Dysfunction in Type 1 Diabetes
Source: Int J Mol Sci. 2026 Aug 19;27(16):7423. doi: 10.3390/ijms27167423 (PMC13513957; doi:10.3390/ijms27167423)
Supplement: Supplementary file 1 [file ijms-27-07423-s001.zip › ijms-4406279-supplementary.pdf]

Table S1. List of all the used antibodies.

Antibody	Molecular Weight (kDa)	WB Dilution	IF Dilution	Source
β-Actin	42	1:5000	1:100	Elabscience Biotechnology, Wuhan, China# E-AB-20031
F-ACTIN	42	1:1000	1:100	Thermo Fisher Scientific, Waltham, MA, USA # MA1-80729
EPS8	97	1:2000	1:100	Thermo Fisher Scientific, Waltham, MA, USA # PA5-21295
FSCN-1	55	1:1000	-	Thermo Fisher Scientific, Waltham, MA, USA # MA5-11483
ARP2	45	1:3000	-	ABclonal Science, Inc. Woburn, MA, USA # A5734-100
ARP3	47	1:500	-	Thermo Fisher Scientific, Waltham, MA, USA # PA5-17288
N-WASP	55	1:1000	-	ABclonal Science, Inc. Woburn, MA, USA #A2576-100
FZD5	65	1:2000	-	ABclonal Science, Inc. Woburn, MA, USA # A12775
p-DVL2 -S143	81	1:1000	-	Biorbyt, Durham, NC, USA # orb156590
DVL2	79	1:3000	-	GeneTex, Irvine, CA, USA # GTX111156
DAAM1	125	1:3000	1:100	Sigma-Aldrich, Milan, Italy # HPA026605
RhoA	21	1:1000	-	Thermo Fisher Scientific, Waltham, MA, USA # MA1-134
ROCK1	158	1:2000	-	Elabscience Biotechnology, Wuhan, China # E-AB-62198
p-LIMK1-T508	73	1:1000	-	ABclonal Science, Inc. Woburn, MA, USA # AP0387
LIMK1	74	1:3000	-	ABclonal Science, Inc. Woburn, MA, USA # A23948
p-CFL1-S3	17	1:2000	1:100	ABclonal Science, Inc. Woburn, MA, USA #AP0178

CFL1	18	1:3000	-	ABclonal Science, Inc. Woburn, MA, USA # A2658
RICTOR	190	1:3000	-	Sigma-Aldrich, Milan, Italy # SAB4200141
p-PKC-T658	76	1:1000	-	Sigma-Aldrich, Milan, Italy # SAB4504096
PKC	76	1:2000	-	Sigma-Aldrich, Milan, Italy # SAB4502354
p-MARCKS-S162	75	1:2000	1:100	ABclonal Science, Inc. Woburn, MA, USA # AP0402
MARCKS	75	1:3000	-	ABclonal Science, Inc. Woburn, MA, USA # A0936
α-TUBULIN	52	1:5000	-	Elabscience Biotechnology, Wuhan, China #E-AB-20036
Goat anti-rabbit IgG HRP	-	1:5000	-	Vector Laboratories, Burlingame, CA, USA # PI-1000
Goat anti-mouse IgG HRP	-	1:5000	-	BioActs, Namdong-gu, Incheon, Korea # RSA1122
Goat anti-rabbit Alexa Fluor 488	-	-	1:500	Thermo Fisher Scientific, Waltham, Ma, USA # A32731
Goat anti-mouse CF™ 568	-	-	1:250	Sigma-Aldrich, Milan, Italy # SAB4600082
PNA lectin Alexa Fluor 568	-	-	1:50	Thermo Fisher Scientific, Waltham, Ma, USA # L32458

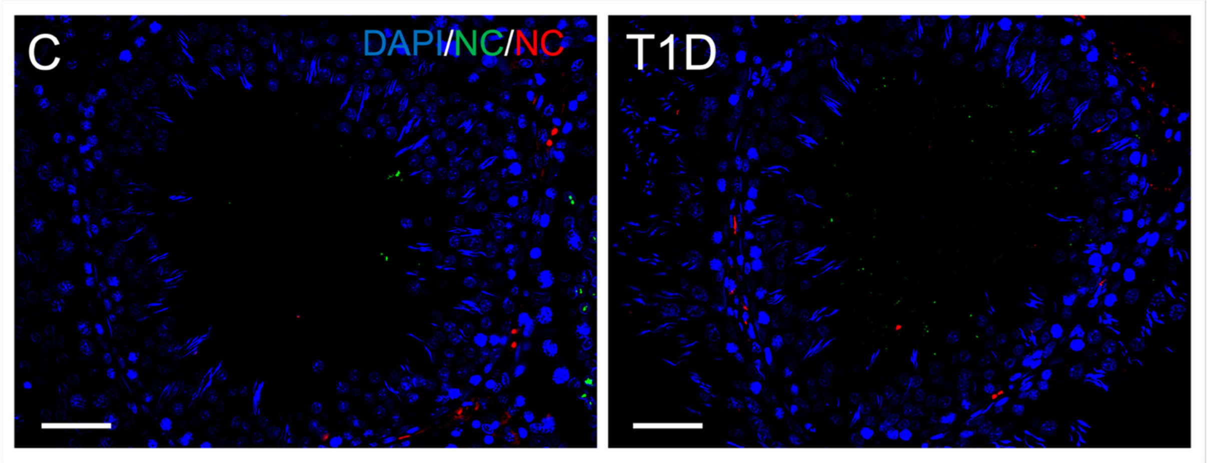

Figure S1. Negative controls for immunofluorescence (IF) staining. Representative IF images of testicular sections from control (C) and type 1 diabetic (T1D) rats processed by omitting the primary antibodies and incubating with the corresponding secondary antibodies. The slides were counterstained with DAPI-fluorescent nuclear staining (blue). Scale bars = 20 μm.
